# Interaction of Inflammation and Hyperoxia in a Rat Model of Neonatal White Matter Damage

**DOI:** 10.1371/journal.pone.0049023

**Published:** 2012-11-14

**Authors:** Felix Brehmer, Ivo Bendix, Sebastian Prager, Yohan van de Looij, Barbara S. Reinboth, Julia Zimmermanns, Gerald W. Schlager, Daniela Brait, Marco Sifringer, Stefanie Endesfelder, Stéphane Sizonenko, Carina Mallard, Christoph Bührer, Ursula Felderhoff-Mueser, Bettina Gerstner

**Affiliations:** 1 Department of Neonatology, Charité University Medical Center, Berlin, Germany; 2 Department of Pediatrics I – Neonatology, University Hospital Essen, Essen, Germany; 3 Department of Pediatrics, University of Geneva, Genève, Switzerland; 4 Laboratory of Functional and Metabolic Imaging, Ecole Polytechnique Fédérale de Lausanne, Lausanne, Switzerland; 5 Department of Anesthesiology and Intensive Care Medicine, Charité University Medical Center, Berlin, Germany; 6 Department of Neuroscience and Physiology, Sahlgrenska Academy, University of Gothenburg, Gothenburg, Sweden; 7 Department of Pediatric Cardiology, Pediatric Heart Center, Justus-Liebig-University, Gießen, Germany; Massachusetts General Hospital/Harvard Medical School, United States of America

## Abstract

Intrauterine infection and inflammation are major reasons for preterm birth. The switch from placenta-mediated to lung-mediated oxygen supply during birth is associated with a sudden rise of tissue oxygen tension that amounts to relative hyperoxia in preterm infants. Both infection/inflammation and hyperoxia have been shown to be involved in brain injury of preterm infants. Hypothesizing that they might be additive or synergistic, we investigated the influence of a systemic lipopolysaccharide (LPS) application on hyperoxia-induced white matter damage (WMD) in newborn rats. Three-day-old Wistar rat pups received 0.25 mg/kg LPS i.p. and were subjected to 80% oxygen on P6 for 24 h. The extent of WMD was assessed by immunohistochemistry, western blots, and diffusion tensor (DT) magnetic resonance imaging (MRI). In addition, the effects of LPS and hyperoxia were studied in an *in vitro* co-culture system of primary rat oligodendrocytes and microglia cells. Both noxious stimuli, hyperoxia, and LPS caused hypomyelination as revealed by western blot, immunohistochemistry, and altered WM microstructure on DT-MRI. Even so, cellular changes resulting in hypomyelination seem to be different. While hyperoxia induces cell death, LPS induces oligodendrocyte maturity arrest without cell death as revealed by TUNEL-staining and immunohistological maturation analysis. In the two-hit scenario cell death is reduced compared with hyperoxia treated animals, nevertheless white matter alterations persist. Concordantly with these *in vivo* findings we demonstrate that LPS pre-incubation reduced premyelinating-oligodendrocyte susceptibility towards hyperoxia *in vitro*. This protective effect might be caused by upregulation of *interleukin-10* and *superoxide dismutase* expression after LPS stimulation. Reduced expression of transcription factors controlling oligodendrocyte development and maturation further indicates oligodendrocyte maturity arrest. The knowledge about mechanisms that triggered hypomyelination contributes to a better understanding of WMD in premature born infants.

## Introduction

Worldwide, about 12 to 13 million babies are born preterm each year. Meaning the rate of preterm birth is about 9–10% in industrialized countries [1], [2]. Intrauterine infection is a major cause for preterm birth [3], [4]. In addition, compared to intrauterine conditions birth is associated with exposure to relative hyperoxia by a dramatic rise of the oxygen tissue tension [5]. Furthermore, respiratory distress of preterms is treated with nonphysiologic oxygen-supply [6]. Experimental and clinical data suggest that both inflammation [7]–[9] and elevated oxygen concentrations [6], [10]–[12] contribute to brain injury of preterm infants. Even though this two-hit hypothesis of first infection/inflammation followed by hyperoxia reflects the clinical conditions of many preterm infants, previous research on the combination of hyperoxia and inflammation only focused on cardiovascular diseases [13].

Lipopolysaccharide (LPS) is widely used to induce an inflammatory response in animal models of neonatal brain injury [14], [15]. LPS-induced brain-inflammation is characterized by pro-apoptotic cytokine release and oxidative/nitrosative stress [16]–[21]. These inflammatory mediators trigger strong alterations of oligodendroglia (OL) survival and development, leading to hypomyelination [22]–[25].

In previous studies, our group demonstrated a maturation dependent OL cell death induced by hyperoxia (80% oxygen). The hyperoxia-induced apoptosis in the developing rodent brain is induced by intrinsic [26] and extrinsic [27] pathways, and is triggered by pro-inflammatory cytokines [28], induction of matrix metalloproteinases [29] and oxidative/nitrosative stress [30], [31]. Furthermore, *in vitro* data demonstrate that immature oligodendrocytes (O4^+^, O1^−^, MBP^−^; pre-OL) are highly susceptible to elevated oxygen levels and undergo caspase-dependent cell death, whereas mature oligodendrocytes (O4^+^, O1^+^, MBP^+^) show less susceptibility to hyperoxia [32], [33]. *In vivo*, rat pups exposed to 80% oxygen for 24 h at postnatal day 3 (P3) or P6 but not at P10 exhibit an intense loss of myelin basic protein (MBP) at P11 [33]. Hyperoxia induces a disruption of OL development, with a profound loss of NG2^+^ O4^−^ oligodendrocyte progenitor cells (OPC) and a diminished population of mature oligodendrocytes (CC1^+^) in neonatal mice. Although differences in oligodendroglia cell populations are compensated and MBP expression increases to control level after a four day period of recovery, differences in MBP microstructure, detected by lower fractional anisotropy (FA) in diffusion tensor imaging (DTI), persist up to P60 [11].

The role of inflammation as an effector on a second insult is being intensely discussed in other models of neonatal brain injury, such as hypoxia-ischemia or excitotoxicity, and can result in sensitization [7], [23], [34]–[36] as well as tolerance [37]–[39] depending on the severity of insult and temporal distance. However, the influence of infection/inflammation on hyperoxia-induced apoptosis in the developing brain, specifically with regard to pre-OL survival or death, remains elusive.

To shed more light on the crosstalk between inflammation and hyperoxia, we analyzed apoptosis, oligodendrocyte maturation, myelination and white matter microstructure in neonatal rat brains after induction of systemic inflammation and exposure to high oxygen levels. We further determined cell death, pro- and anti-inflammatory gene expression and oligodendrocyte maturation in an oligodendrocyte-microglia-co-culture-model. *In vivo* we found a disrupted oligodendrocyte maturation correlating with hypomyelination in LPS stimulated rat pups, whereas hyperoxia-triggered hypomyelination can be attributed to cell death. Hyperoxia exposed rat pups and rat pups underlying the two-hit scenario did not show significant differences in myelin basic protein expression and white matter microstructure, but the number of dying oligodendrocytes is reduced in the two-hit scenario. Furthermore we provide evidence that LPS pre-treatment reduces pre-OL susceptibility towards hyperoxia-induced cell death and detected morphological changes and developmental deficits *in vitro*. Interestingly, we found an increased gene expression of the anti-inflammatory cytokines *interleukin-10* (*IL-10*) and the reactive oxygen species scavenger *superoxide dismutase* (*SOD2*) after LPS stimulation, possibly involved in reduced oligodendrocyte susceptibility against hyperoxia.

## Materials and Methods

### Animals and experimental procedure

All animal experiments were performed according to institutional guidelines of the Charité-Universitätsmedizin Berlin, Germany or the University Hospital Essen, Germany.

3-day-old (P3) Wistar rat pups (BgVV, Berlin, Germany) were randomly distributed in four groups and treated as follows: (1) VN: vehicle (normal saline) i.p. injection and normoxia (21% O_2_); (2) LN: 0.25 mg/kg LPS (*E.coli* O55:B5, Sigma, Steinheim, Germany) i.p. injection and normoxia; (3) VH: vehicle i.p. injection and hyperoxia (80% O_2_); (4) LH: 0.25 mg/kg LPS i.p. injection and hyperoxia. All rat pups were kept at room air until P6. Animals of group 3 and 4 were placed together with their dam in a computer-controlled oxygen-chamber (OxyCycler, BioSpherix, Lacona, NY) for 24 h containing an oxygen-enriched atmosphere (80% O_2_). Animals belonging to group 1 and 2 stayed at room air with another dam. Additionally, we stimulated rat pups at the same point in time (P6) with LPS and hyperoxia using above described experimental design. General health of the rat pups was monitored daily for food intake (filling of the stomach with milk) and bodyweight was determined at P3, P6, P7, and before sacrifice. The rat pups were killed by receiving an overdose of chloral hydrate, either directly after hyperoxia exposure at P7, or at P11 and P21. Gender was determined by anatomical differences and sex matched distribution was found for all groups. For protein collection, pups were transcardially perfused with PBS, the olfactory bulb and the cerebellum were removed and the brain hemispheres were snap-frozen in liquid nitrogen and stored at −80°C until further analysis. For histological studies the pups were transcardially perfused with PBS followed by 4% paraformaldehyde. The brains were postfixed for 24 h with 4% paraformaldehyde and then cryoprotected with 30% sucrose in PBS.

### Ethics statement

This study and all animal experiments were approved by the local ethics committee (North Rhine-Westphalia State Environment Agency), ethical permit number G1111/10 and carried out in accordance with local institutional guidelines.

### Immunoblotting

Snap-frozen tissue was homogenized in ice-cooled radioimmunoprecipitation assay (RIPA) buffer containing protease inhibitor. The homogenate was centrifuged at 1.050 *g* (4°C) for 10 min, and the microsomal fraction was subsequently centrifuged at 17.000 *g* (4°C) for 20 min. The protein concentration was determined by using a BCA kit (Thermo Fisher Scientific, Rockford, IL). Twenty micrograms of the resulting cytosolic protein extracts were heat denaturated in Laemmli sample loading buffer, separated by 15% sodium dodecyl sulfate polyacrylamide gel electrophoresis, and electrotransferred onto a nitrocellulose membrane (0.2 µm pore, Protran; Schleicher & Schüll, Dassel, Germany). Equal loading and transfer of proteins was confirmed by staining the membranes with Ponceau S solution (Fluka, Buchs, Switzerland). 5% nonfat dry milk was used for blocking nonspecific protein binding in Tris-buffered saline/0.1% Tween 20 (TBST). Membranes were incubated over night (4°C) with primary antibody monoclonal rabbit anti-cleaved caspase-3 (1∶1.000, Cell Signaling, Danvers, MA) or monoclonal mouse anti-MBP (1∶5.000, Abcam, Cambridge, UK) in 5% nonfat dry milk in TBST. Horseradish-peroxidase-conjugated secondary antibodies (anti-rabbit and anti-mouse, Dako, Glostrup, Denmark) were diluted 1∶1000 in 1% BSA in TBST. Chemiluminescent detection was performed using enhanced chemiluminescence (ECL; Amersham Biosciences, Bucks, UK). For visualization and densitometric analysis ChemiDoc XRS+ imaging system and ImageLab software (Bio-Rad, Munich, Germany) was used. For stripping, membranes were incubated with Roti-Free (Carl Roth, Karlsruhe, Germany) at 56°C for 30 min, then washed, blocked and reprobed overnight at 4°C with monoclonal mouse anti-β-actin antibody (1∶10.000; Sigma).

### Immunohistochemistry

Myelination was evaluated by immunohistochemistry in rats sacrificed at P11 on serial 16 µm coronal cryosections, as previously described [40]. Sections were blocked and incubated overnight with primary antibody monoclonal mouse anti-MBP (1∶1.000, SMI-99, Sternberger Monoclonals, Baltimore, MD), rinsed, and then incubated with the appropriate secondary antibody (Alexa Fluor 594; Invitrogen, Carlsbad, CA) for 1 h at room temperature. Changes in MBP expression were used as a marker for white matter injury [33], [40].

To determine differences in oligodendroglia maturation O4 (1∶100, monoclonal mouse anti-O4, Jackson Laboratories, Baltimore, PA) and APC-CC1 (1∶100, monoclonal mouse anti-APC, Calbiochem, Darmstadt, Germany) staining was performed on 12 µm coronal cryosections in rats sacrificed at P7. Sections were blocked and incubated overnight with primary antibody, rinsed, and then incubated with the appropriate secondary antibody (1∶500, Alexa Fluor 594; Invitrogen) for 1 h at room temperature. Nuclear staining was made by a 5 min DAPI (10 ng/ml, Invitrogen) incubation. For each animal and staining four images were taken in the white matter region. Images were counted by two independent blinded observers.

Terminal deoxynucleotidyl transferase-mediated biotinylated UTP nick end labeling (TUNEL) assays were performed on 12 µm coronal cryosections according to the manufacturer's instructions (Calbiochem) and degenerating cells were determined between bregma level −1.8 and −4.5 in the white matter, thalamus and cortex, regions that are highly affected in neonatal brain injury [41]–[44]. For determination of oligodendrocyte cell death sections were co-stained with Olig2 (1∶200, Millipore) followed by TUNEL (Roche, Mannheim, Germany). For each animal and region four images were taken and counted by two independent blinded observers.

### Diffusion Tensor–Magnetic Resonance Imaging (DT-MRI)

DT-MRI acquisition was performed at two different points in time, i.e. P11 and P21. All experiments were performed on an actively-shielded horizontal 9.4 T/31 cm magnet (Varian/Magnex) equipped with 12-cm gradient coils (400 mT/m, 120 µs) with a transmit-receive 25 mm birdcage RF coil. After manual adjustment of the first and second order shims (water linewidth ∼20 to 40 Hz) spin-echo sequence with addition of the Stejskal-Tanner diffusion gradients was used. Diffusion gradients were applied along twelve spatial directions: dual gradient diffusion gradient sampling scheme [45] as well as the six opposite directions to cancel cross terms [46]. Intensity, duration and diffusion time were set to 22 G/cm, 3 ms and 20 ms respectively, given a b-value of 1185 s/mm^2^. A field of view of 16×16 mm^2^ was sampled on a 128×64 cartesian grid. Multi-slice DT images were acquired (12 slices of 0.5 mm thickness) in the axial plane with 10 averages and TE/TR = 30/2000 ms. Using an in house Matlab script (Mathworks, Natick, MA), diffusivity values (D//and D⊥) as well as fractional anisotropy (FA) was derived from the tensor. The program allows manual delineation of region of interest (ROI) on the FA maps. Four different regions of the brain were analyzed: the corpus callosum (CC), the external capsule (EC), the superficial layer of sensori-motor cortex (SCx) and the basal ganglia (BG).

### Primary oligodendrocyte-microglia-co-culture

Primary OL and microglia cells (MG) were prepared from the cerebral hemispheres of Sprague Dawley rats at P1 to P2 using a shaking method of mixed-glia-cultures [47] with modifications, as described previously [48]. Purified OL were cultured in 24- or 48-well plates with a density of 20.000 cells/cm^2^ for 3 days in a serum-free basal-defined medium (BDM: DMEM (Invitrogen), 0.1% bovine serum albumin (Sigma), 50 µg/ml human apo-transferrin (Sigma), 50 µg/ml insulin (Sigma), 30 nM sodium selenite (Sigma), 10 nM D-biotin (Sigma), 10 nM hydrocortisone (Sigma), 10 ng/ml human recombinant platelet-derived growth factor (Peprotech, Rocky Hill, NJ), and 10 ng/ml human recombinant basic fibroblast growth factor (Peprotech) before MG were added. Cultures were kept in a humidified incubator at 37°C and 5% CO_2_. Purified OL contained <5% astrocytes and <1% microglia and purified microglia contained <0.1% astrocytes and <1% oligodendrocytes. OL-MG-ratio was set to 1∶1 following cell count results in the rat brain [49]. Next day media was changed using BDM with or without 1 µg/mL LPS. After 24 h pre-incubation cultures were exposed to 80% oxygen for another 8 hours or stayed at standard growth conditions. Additionally, co-cultures were co-exposed to LPS at the beginning of the experiment and/or hyperoxia for 8 h. Supernatants out of 24-well plates was collected, centrifuged and used for cytotoxicity assay. Co-cultures grown in 6-well plates were used for RNA isolation.

### Cytotoxicity assay

Lactate dehydrogenase (LDH) activity was quantified in 50 µl samples of medium supernatant, using a colorimetric cytotoxicity assay kit according to the manufacturer instructions (Roche, Mannheim, Germany). Absorbance data were obtained using an Infinite M200 plate reader (Tecan, Salzburg, Austria) at 492 nm wavelength, and 650 nm as reference wavelength. After blank subtraction data were expressed as fold change to control culture values.

### Gene expression analysis

Total cellular RNA was isolated at an early point in time (4 h after LPS stimulation) and at a late point in time (24 h after LPS stimulation) by acidic phenol/chloroform extraction and DNase I treatment (Qiagen, Hilden, Germany). Subsequently, 1 µg of RNA was reverse transcribed at 42°C for 1 h with 200 U of Moloney murine leukemia virus reverse transcriptase and random-primer (Promega, Madison, WI). The PCR products were quantified in real-time, using dye-labeled fluorogenic reporter oligonucleotide. Primer sequences are given in Table 1. *Hypoxanthine-guanine phosphoribosyl-transferase* was used as internal standard. Real-time PCR was performed in duplicates and repeated two times for each sample using a total reactive volume of 11 µl which contained 5 µl of 2× KAPA PROBE FAST qPCR Mastermix (PEQLAB Biotechnologie GMBH, Erlangen, Germany), 2,5 µl of 2 µM oligonucleotide mix (forward and reverse primer, BioTeZ, Berlin, Germany), 0,5 µM probe, and 20 ng of cDNA template. The PCR amplification was performed with the StepOnePlus™ Real-Time PCR System (Applied Biosystems, Foster City, CA; 40 cycles with each cycle at 94°C for 15 sec and 60°C for 1 min) and fluorescent data were converted into cycle threshold (CT) values. Target levels were normalized to *HPRT* levels and data were expressed as fold change of control using ΔΔCt-method.

**Table 1:** Primer and probe sequences.

**TNFα**	NM_012675.3	forward	CCCCCAATCTGTGTCCTTCTAAC
		reverse	CGTCTCGTGTGTTTCTGAGCAT
		probe	TAGAAAGGGAATTGTGGCTC
**IL-1β**	NM_031512.2	forward	CTCCACCTCAATGGACAGAACA
		reverse	CACAGGGATTTTGTCGTTGCT
		probe	CTCCATGAGCTTTGTACAAG
**IL-10**	NM_012854.2	forward	GAAGACCCTCTGGATACAGCTGC
		reverse	TGCTCCACTGCCTTGCTTTT
		probe	CGCTGTCATCGATTTCTCCCCTGTGA
**SOD2**	NM_017051.2	forward	GACCTACGTGAACAATCTGAACGT
		reverse	AGGCTGAAGAGCAACCTGAGTT
		probe	ACCGAGGAGAAGTACCACGA
**Sox9**	XM_001081628.2	forward	CGGAGGAAGTCGGTGAAGAA
		reverse	TGCAGCGCCTTGAAGATG
		probe	ACAGACTCACATCTCTCCTA
**Sox10**	NM_019193.1	forward	CCGCACCTCCACAATGCT
		reverse	GGTACTTGTAGTCCGGATGGTCTTT
		probe	TTGCTGAACGAGAGTGACAA
**CNP**	NM_012809.1	forward	GGCGTGCTGCACTGTACAAC
		reverse	AAGATCTCCTCACCACATCCTGTT
		probe	AATTCTGTGACTACGGGAAG
**MBP**	NM_001025291.1	forward	GAGCCCTCTGCCTTCTCATG
		reverse	AGGGAGCCGTAGTAGTGGGTAGTTC
		probe	ACATGTACAAGGACTCACAC
**HPRT**	NM_012583.3	forward	GGAAAGAACGTCTTGATTGTTGAA
		reverse	CCAACACTTCGAGAGGTCCTTTT
		probe	CTTTCCTTGGTCAAGCAGTACAGCCCC

### Statistical analysis

Values are presented as mean+SEM. The significance of differences among groups was determined by analysis of variance (one-way ANOVA) followed by *post hoc* independent sample *t*-test multiple comparisons. Adjusted *P*-values of <0.05 were considered statistically significant.

## Results

To investigate the influence of inflammatory pre-treatment on hyperoxia-induced white matter damage, Wistar rat pups were stimulated with 0.25 mg/kg LPS or vehicle i.p. on P3 and were subjected to 21% or 80% oxygen on P6 for 24 hours (group declaration: VN: vehicle and normoxia, LN: LPS and normoxia, VH: vehicle and hyperoxia, LH: LPS and hyperoxia).

### LPS application and hyperoxia induce effector caspase-3 in the immature rat brain

**Figure 1:**
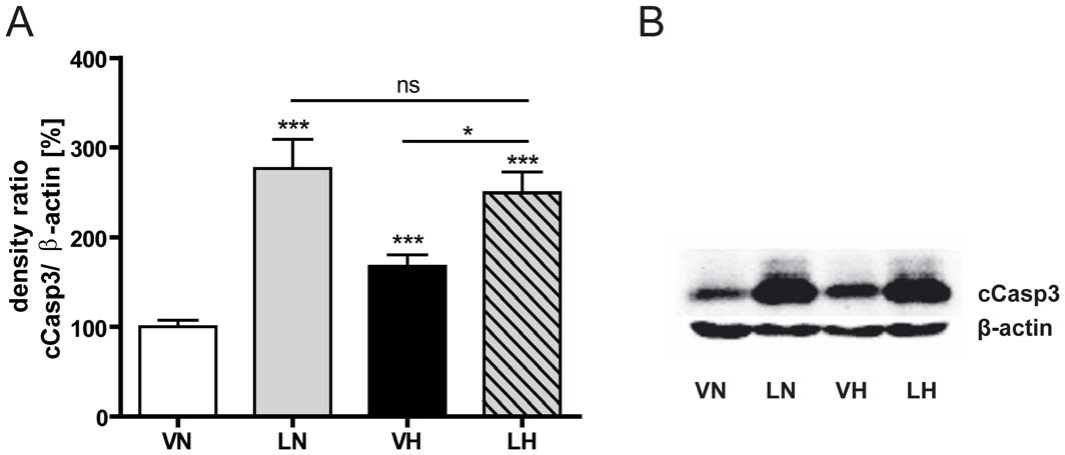
Inflammation and hyperoxia increases cCasp3 expression. Systemic LPS application as well as exposure to 80% oxygen increases caspase-3 activation in the developing rat brain. Results of densitometric western blot quantification (A) and a representative western blot series are shown (B). Animals receiving the two-hit scenario (LH, dashed bar) showed neither a higher nor a faintly cCasp3 expression compared to LPS single-hit treated pups (LN, grey bar). Brain hemisphere extracts were used for western blot analyses with n = 9–11 animals per group. Western blot values represent mean+SEM normalized ratios of the cCasp3 bands to β-actin as an internal standard and VN group was set to 100%. * p<0.05, *** p<0.001 and ns p>0.05; VN: vehicle+normoxia, LN: LPS+normoxia, VH: vehicle+hyperoxia, LH: LPS+hyperoxia.

For evaluation of brain injury after an inflammatory stimulus followed by hyperoxia, apoptotic cell death was measured by determination of cleaved caspase-3 (cCasp3) protein expression on P7, directly after hyperoxia. As shown in Fig. 1, in the single-hit scenarios, LPS (LN: 277% of VN, n = 9, p<0.001, grey bar) or hyperoxia (VH: 168% of VN, n = 10, p<0.001, black bar) led to a significant caspase-3 activation, in which LPS treatment resulted in a more prominent cCasp3 protein expression. In the two-hit condition (LH: 249% of VN, n = 12, p<0.001, dashed bar) cCasp3 protein expression was significantly higher than in pups, treated with hyperoxia alone (p<0.05), but no significant differences (p = 0.59) were detectable between animals receiving LPS with or without additional hyperoxia treatment.

### LPS application and hyperoxia decrease myelin basic protein expression in the developing rat brain

**Figure 2:**
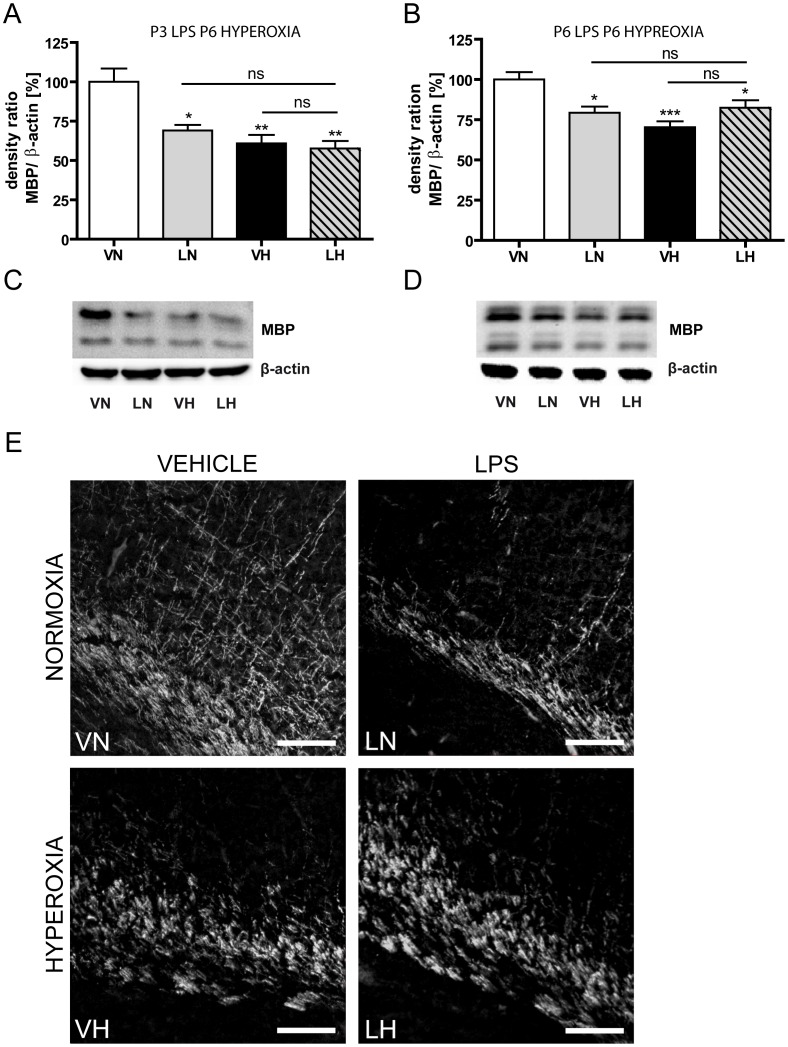
Inflammation and hyperoxia decreases MBP expression. A significantly decrease of MBP protein expression as a sign of hypomyelination occurs in all experimental settings with similar severity. Results of densitometric western blot quantification of P3 LPS P6 hyperoxia experiment (A), results of densitometric western blot quantification of P6 LPS P6 hyperoxia experiment (B), a representative western blot series of P3 LPS P6 hyperoxia experiment (C), a representative western blot series of P3 LPS P6 hyperoxia experiment (D) and images of immunohistological stained sections against MBP (E) are shown. Immunohistological staining against MBP supports these findings and illustrates a profound reduction of MBP in external capsule with loss of processes and thinning up to fragmentation of the capsule in all treated groups. Scale bar = 100 µm. Brain hemisphere extracts were used for western blot analyses with n = 9–11 animals per group. Western blot values represent mean+SEM normalized ratios of the MBP bands to β-actin as an internal standard and VN group was set to 100%. * p<0.05, ** p<0.01, *** p<0.001 and ns p>0.05; VN: vehicle+normoxia, LN: LPS+normoxia, VH: vehicle+hyperoxia, LH: LPS+hyperoxia.

Since the final consequence of impaired oligodendroglia is the disturbance of myelination [11], [32], [33], we investigated the degree of myelin basic protein (MBP) expression four days after hyperoxia exposure (P11) in rat pups treated at P3 with LPS and at P6 with hyperoxia (Fig. 2A) as well as in the time matched application (Fig. 2B). As revealed by western blot analysis (Fig. 2A), all treated groups exhibited a decrease of MBP expression. LPS application resulted in the slightest decrease (LN: 69.1%, n = 7, p<0.05, grey bar) whereas hyperoxia and the two-hit scenario exhibited a more drastic reduction of MBP expression (VH: 60.9%, n = 8, p<0.01, black bar; LH: 57.6%, n = 7, p<0.01, dashed bar). However, there was no significant difference between the treated groups. Comparable results were obtained for the time matched scenario (Fig. 2B). MBP expression is reduced significantly (LN: 79%, n = 8, p<0.05, grey bar; VH: 70%, n = 8, p<0.001, black bar; LH: 82%, n = 8, p<0.001, dashed bar). Representative western blots are shown in Fig. 2C for the LPS pre-treatment experiment and in Fig. 2D for the co-exposure experiment. Immunohistological MBP-staining supported these findings and illustrated a profound reduction of MBP-fibers in the WM with loss of processes and thinning up to fragmentation of the capsule in all treated groups (Fig. 2E). At P21 MBP expression was compensated in all groups (Fig. S1).

### LPS application and hyperoxia change WM microstructural integrity

**Figure 3:**
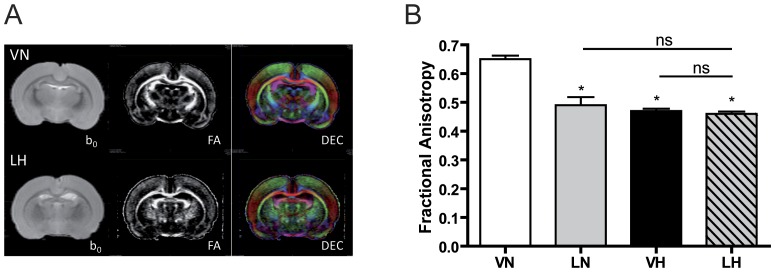
Inflammation and hyperoxia decreases fractional anisotropy in corpus callosum. Typical DT images are presented in (A) T_2_W images (b0), fractional anisotropy (FA) and direction encoded color maps (DEC) of a VN (upper panel) and LH (lower panel) rat brains are shown. (B) Significant decrease of FA was observed in the corpus callosum of the injured groups (LN, VH, LH) compared with control group (VN) due to a significant increase of D⊥ (i.e. myelination defect). There were no significant differences between the injured groups providing evidence of similar damage in corpus callosum with or without hyperoxia after LPS challenge. Mean+SEM were presented with n = 6 animals per group. * p<0.05; VN: vehicle+normoxia, LN: LPS+normoxia, VH: vehicle+hyperoxia, LH: LPS+hyperoxia.

Typical DT-MRI images of P21 brains are presented in Fig. 3A. At P11 a non-significant fractional anisotropy (FA) decrease at all rostro-caudal levels of the corpus callosum was observed in the exposed groups (LH, LN and VH, each n = 3) compared to the control group (VN, n = 3) (data not shown). At P21, we observed a significant decrease of FA (Fig. 3B) at the corpus callosum of the treated groups (LN: 0.49, n = 6, p<0.05, grey bar; VH: 0.47, n = 6, p<0.05, black bar; LH: 0.46, n = 6, p<0.05, dashed bar) compared with the control group (VN: 0.65, n = 6, white bar) due to a significant increase of D⊥ (diffusion perpendicular to the main direction of water diffusion along the WM tracts). For both points in time, there were no significant differences between the treatment groups.

### Hyperoxia triggers oligodendrocyte death whereas LPS disrupts oligodendrocyte maturation

**Figure 4:**
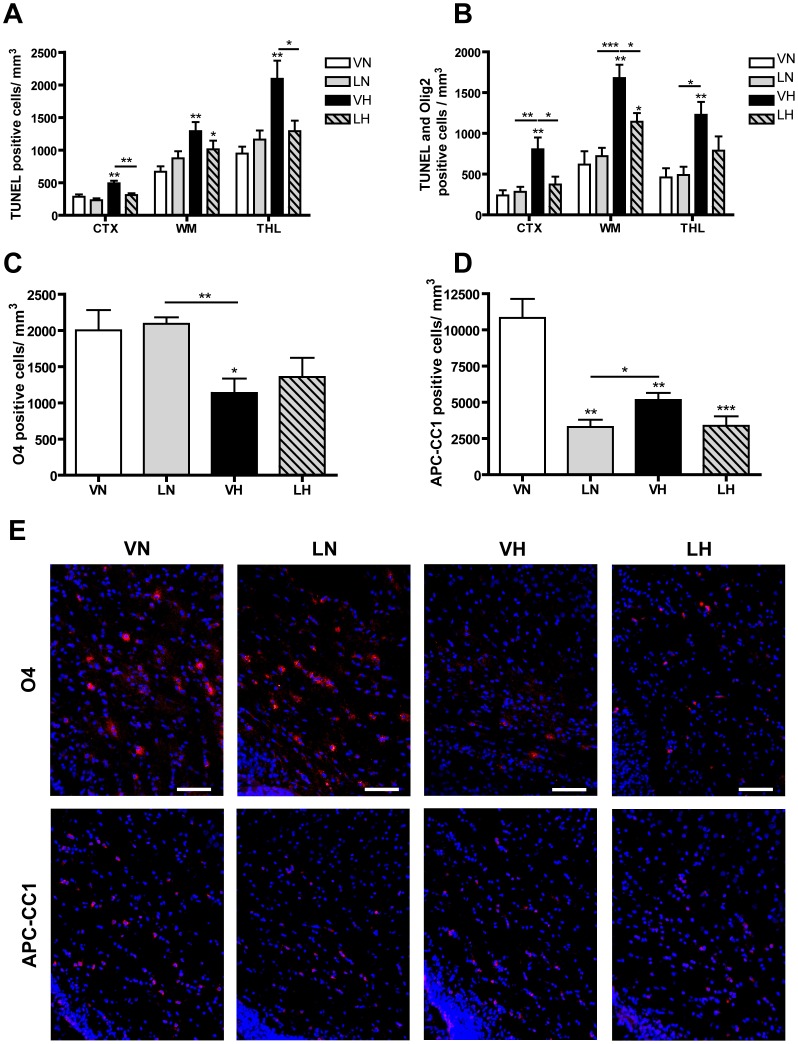
Immunohistological analysis of cell death and oligodendrocyte maturation. Hyperoxia but not LPS induce oligodendrocyte cell death revealed by TUNEL-staining. (A) LPS pre-treatment prior to hyperoxia reduce number of TUNEL-positive cells in CTX, WM and THL (LH, dashed bars). LPS application slightly increases number of TUNEL-positive cells in WM and THL but not in CTX (LN, grey bars), whereas hyperoxia affects all probed brain regions strongly (VH, black bars). TUNEL-positive cells were counted by two blinded observers in 5 sections per animal and 4 randomly assigned fields of each region per section. (B) A co-labeling with Olig2 and TUNEL shows that LPS do not induce oligodendrocyte death (LN, grey bar). Hyperoxia induces oligodendrocyte death (VH, black bar) and LPS pre-treatment reduces hyperoxia induced cell death (LH, dashed bar). (C) Hyperoxia (VH, black bar) but not LPS (LN, grey bar) causes a loss of O4-positive cells. Pups underlying the two-hit scenario exhibit a decreasing tendency of O4-positive cells (LH, dashed bar). (D) In all treated groups the number of APC-CC1-positive cells is decreased. (E) Representative images of O4- and APC-CC1 staining are shown. Scale bar = 100 µm. Mean+SEM were presented as TUNEL-positive cells/mm^3^ with n = 9–11 animals per group. * p<0.05, ** p<0.01and *** p<0.001; VN: vehicle+normoxia, LN: LPS+normoxia, VH: vehicle+hyperoxia, LH: LPS+hyperoxia.

In order to analyze cell death in a region and cell type specific manner we performed immunohistochemical examinations using a co-labeling of Olig2 and TUNEL staining. We counted all TUNEL-positive cells (Fig. 4A) and only TUNEL-positive oligodendrocytes (Fig. 4B) in the following regions: cerebral cortex (CTX), white matter (WM) and thalamus (THL). These areas are known to be most affected in encephalopathy of prematurity [41]–[44].

In the CTX (Fig. 4A) LPS stimulation with or without additional hyperoxia-exposure did not significantly alter number of TUNEL positive cells/mm^3^ (VN: 284, n = 12, white bar; LN: 231, n = 10, p = 0.27, grey bar; LH: 309, n = 12, p = 0.60, dashed bar) whereas hyperoxia results in an increased number of TUNEL-positive cells (VH: 492, n = 11, p<0.01, black bar). In WM regions hyperoxia-treated pups showed a more drastic increase in TUNEL-positive cells compared to untreated rats (VN: 667, n = 12, white bar; VH: 1290, n = 10, p<0.001, black bar). LPS application alone shows an increasing tendency (LN: 874, n = 10, p = 0.15, grey bar) and the LPS application with additional hyperoxia leads to a slight increase in degenerated cells (LH: 1011, n = 12, p<0.05, dashed bar). Differences between the treated groups were not significant. Analogous results were detected in the thalamic region. The strongest increase of TUNEL-positive cells was counted after exposure to 80% oxygen (VN: 946, n = 12, white bar; VH: 2092, n = 11, p<0.01, black bar) and comparatively fewer cells were found after LPS treatment with or without additional hyperoxia exposure (LN: 1161, n = 10, p = 0.24, grey bar; LH: 1290, n = 12, p = 0.55, dashed bar).

Analysis of oligodendrocyte cell death (Fig. 4B) revealed that hyperoxia but not LPS stimulation strongly induces oligodendrocyte death in all regions investigated (CTX: VN: 239, n = 5; LN: 282, p = 0.63; VH: 804, n = 6, p<0.01; LN: 372, n = 6, p = 0.29; WM: VN: 709, n = 5; LN: 720, n = 6, p = 0.60; VH: 1679, n = 6, p<0.01; LH: 1141, n = 6, p<0.05; THL: VN: 458, n = 5; LN: 488, n = 6, p = 0.85; VH: 1227, n = 6, p<0.01; LH: 787, n = 6, p = 0.15). Interestingly, we detected reduced numbers of TUNEL-positive cells in rat pups stimulated with LPS before hyperoxia (VH vs. LH CTX: p<0.05; WM: p<0.05; THL: p = 0.10).

Since we detected hypomyelination without oligodendrocyte cell death in LPS treated pups we hypothesize alterations in the differentiation of oligodendrocytes. As demonstrated by immunohistological analysis of immature oligodendrocytes (O4) and mature oligodendrocytes (APC-CC1) we detected distinct modulations in these oligodendrocyte differentiation markers. In the white matter, the origion of oligodendrocyte progenitors, we found a loss of O4-positive cells after hyperoxia which was not detected after LPS treatment (Fig. 4C: VN: 2001, n = 5; LN: 2090, n = 5, p = 079; VH: 1136, n = 5, p<0.05; LH: 1356, n = 5, p = 0.13). In the two-hit scenario, the number of O4-positive cells was reduced, however this reduction failed to reach statistical significance. Interestingly, the number of mature oligodendrocytes as revealed by APC-CC1 staining was decreased in all treated groups (Fig. 4D: VN: 10823, n = 5; LN: 3297, n = 5, p<0.01; VH: 5163, n = 5, p<0.01; LH: 3380, n = 5, p<0.001). Representative images of O4 and APC-CC1 staining are shown in Fig. 4E.

### LPS application and hyperoxia increase cytotoxicity of oligodendrocyte–microglia–co-cultures and change morphology of oligodendrocytes

In order to elucidate the results in a more cell-specific scenario, we established an oligodendrocyte–microglia–co-culture-system to investigate the effect of LPS pre-incubation on hyperoxia induced cell death. Primary oligodendrocyte precursor cells were cultured until they expressed O4^+^O1^−^MBP^−^ maturity level. Then, primary microglia cells, with the same density, were added to the culture. LPS-activated microglia cells are a potent source for cytokines and reactive oxygen species, which influence the maturity and survival of immature oligodendrocytes [18], [21], [50]. Oligodendrocyte–microglia–co-cultures were treated with or without 1 µg/ml LPS for 24 h followed by 8 h exposure to 80% oxygen or incubation under standard growth conditions. Lactate dehydrogenase (LDH) release was used to quantify cytotoxicity.

**Figure 5:**
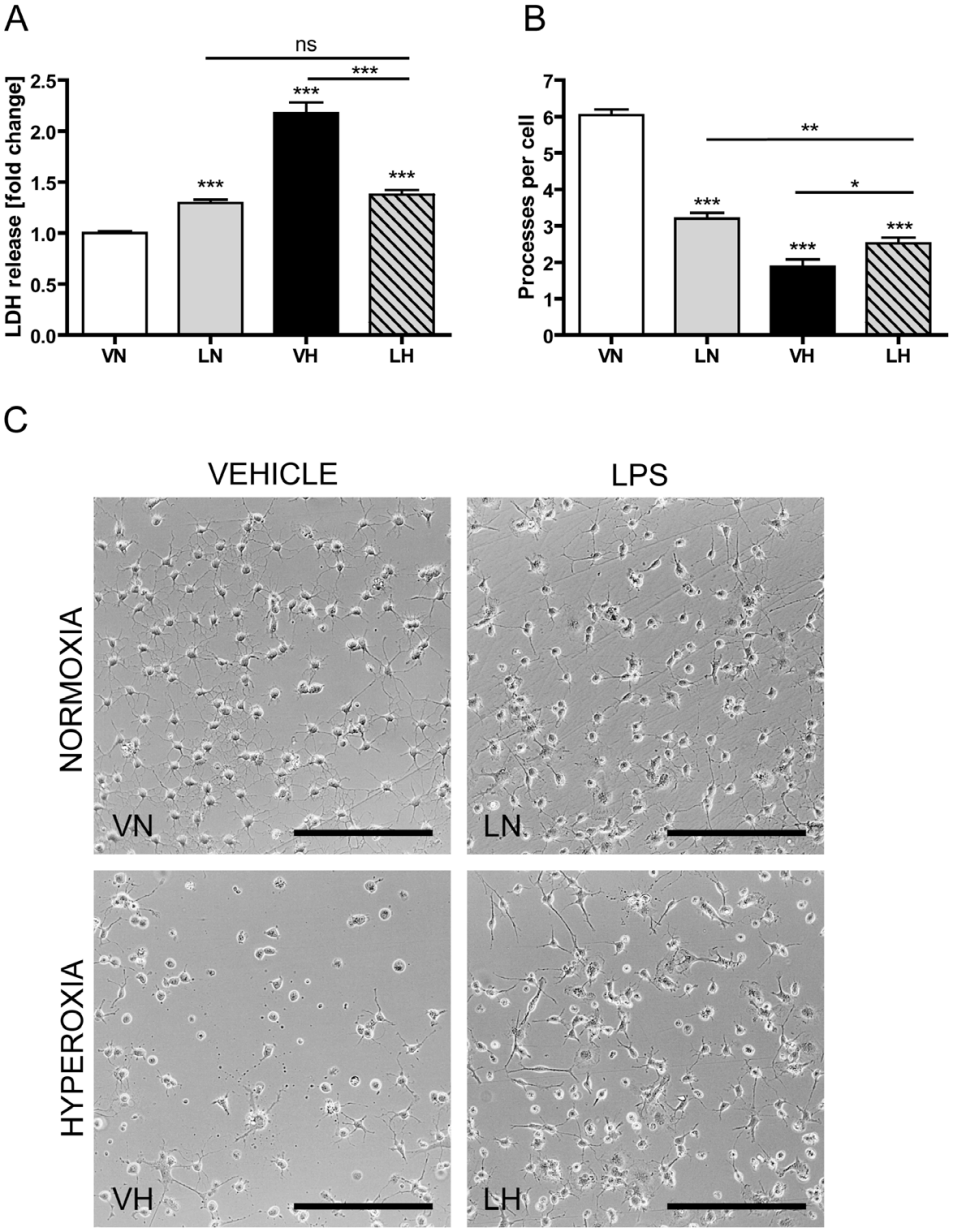
LPS pre-treatment reduces pre-OL susceptibility towards hyperoxia. Primary oligodendrocyte-microglia-co-cultures were stimulated with LPS 24 h before exposure to 80% oxygen for 8 h. Results of a cytotoxicity assay, based on LDH release (A), data of morphological analysis by counting of processes per cell (B) and representative images of the oligodendrocyte-microglia-co-cultures are shown (C). LPS stimulated co-cultures have a slight increase in LDH release but a distinct loss of processes per cell (LN, grey bars) whereas hyperoxia induces profound cell death and numerous cells detached from the culture plate (VH, black bars). Co-cultures exposed to the two-hit scenario are more resistant against hyperoxia (LH, dashed bars). Five independent experiments were used for data collection and means+SEM were shown. One image out of the center of the well was used for counting processes for each group and experiment. Scale bar = 200 µm, * p<0.05, ** p<0.01, *** p<0.001 and ns p>0.05; VN: vehicle+normoxia, LN: LPS+normoxia, VH: vehicle+hyperoxia, LH: LPS+hyperoxia.

As shown in Fig. 5A, LPS stimulation caused a 1.3 fold increase in LDH release (LN: 1.3, p<0.001, grey bar), whereas purified cultures of oligodendrocytes or microglia alone do not release LDH after LPS administration (data not shown). Hyperoxia treated co-cultures exhibited the highest LDH release with a 2.2 fold increase (Fig. 5A, VH: 2.2, p<0.001, black bar). Interestingly, co-cultures exposed to the two-hit scenario were less susceptible to hyperoxia induced cell death. LDH release of co-cultures treated with LPS and hyperoxia was 1.4 fold higher compared to control-cultures (Fig. 5A, LH: 1.4, p<0.001, dashed bar), but significantly lower compared to hyperoxia treated co-cultures (P<0.001). There was no significant difference in LDH-release between LPS treated co-cultures and co-cultures exposed to the two-hit scenario (LN vs. LH, p = 0.197).

When co-cultures were stimulated at the same point in time with LPS and hyperoxia for 8 h, LPS alone does not induce LDH release (Fig.S2: LN: 1.02, p = 0.43, grey bar). Hyperoxia exposed co-cultures show the highest LDH release (VH: 1.8, p<0.001, black bar) and the decreased susceptibility of LPS treated co-cultures against hyperoxia is detected in this setting as well (LH: 1.45, p<0.001, dashed bar; VH vs. LH p<0.001).

Phase-contrast-photomicrographs were made to analyze morphological changes by counting the number of processes per cell. Oligodendrocytes of untreated co-cultures had 6.0±0.1 processes per cell (Fig. 5B, VN) and built up a complex network (Fig. 5C,VN), whereas oligodendrocytes of LPS pre-stimulated co-cultures had only 3.2±0.2 processes per cell (Fig. 5B, LN, p<0.001) with a shorter and less branched shape (Fig. 5C, LN). Hyperoxia exposed cells showed a profound loss of processes per cell, 1.9±0.2 (Fig. 5B, VH, p<0.001), or detached completely from the culture plate (Fig. 5C, VH). In the two-hit scenario, cells had 2.5±0.2 processes per cell with a shorter and less branched shape (Fig. 5B, p<0.001 and 5C, LH).

### LPS activated co-cultures express high amounts of pro-inflammatory cytokines at 4 h whereas protective genes dominate at 24 hours

**Figure 6:**
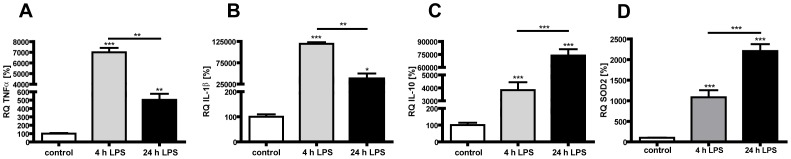
LPS activated microglia cells modulate inflammatory and reactive oxygen scavenger gene expression in a time-dependent manner. Gene expression analysis of co-cultures after 4 and 24 h of LPS-stimulation using quantitative Real-Time PCR show an increased expression of pro-inflammatory genes at 4 h (grey bars) which declines at 24 h (black bars) as shown in (A) for *TNFα* and (B) *IL-1β*. Gene expression of the anti-inflammatory cytokine *IL-10* (C) and the reactive oxygen scavenger *SOD2* is increased at 4 h (grey bars) and increases further at 24 h (black bars). Experiments were performed three times in dublicates. * p<0.05, ** p<0.01 and *** p<0.001; VN: vehicle+normoxia, LN: LPS+normoxia, VH: vehicle+hyperoxia, LH: LPS+hyperoxia.

Since we indicated a LPS-induced tolerance effect in the two-hit scenario, we hypothesized protective mechanisms. Therefore we performed quantitative Real-Time PCR analysis using RNA from co-cultures after 4 and 24 h of LPS stimulation. We found a high expression of the pro-inflammatory cytokines *TNFα* and *IL-1β* 4 h post LPS stimulation which declines at 24 h post LPS stimulation (Fig. 6A: *TNFα* (4 h) 6998%, p<0.001, grey bar; (24 h) 503%, p<0.01, black bar; Fig. 6B: *IL-1β* (4 h) 118465%, p<0.01, grey bar; (24 h) 42406%, p<0.05, black bar). Gene expression of the anti-inflammatory cytokine *IL-10* and the reactive oxygen species scavenger *SOD2* is also increased after 4 h and increases further in the later phase of LPS stimulation (Fig. 6C: *IL-10* (4 h) 3832%, p<0.001, grey bar; (24 h) 73738%, p<0.001, black bar; Fig. 6D: *SOD2* (4 h) 1085%, p<0.001, grey bar; (24 h) 2207%, P<0.001, black bar).

### Oligodendrocytes show an impaired development and maturation in LPS stimulated co-cultures

**Figure 7:**
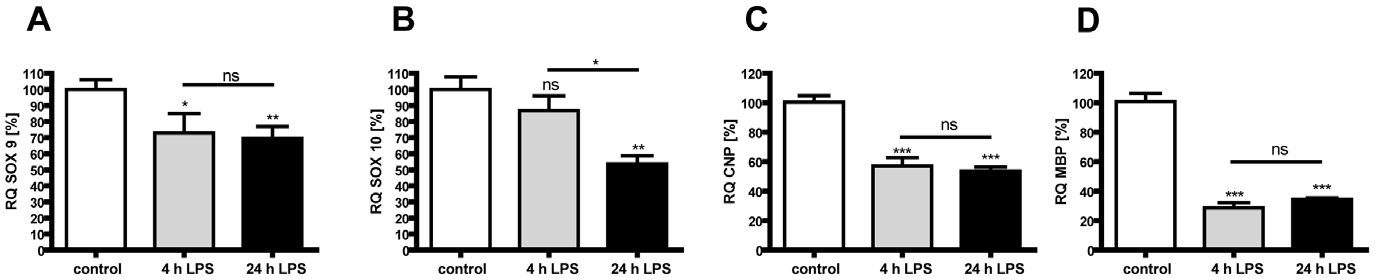
LPS activated microglia decrease oligodendrocyte development and maturation. For evaluation of disturbed oligodendrocyte maturation *in vitro* we did gene expression analysis of co-cultures after 4 and 24 h of LPS-stimulation using quantitative Real-Time PCR. *Sox9* (A), CNP (C) and MPB (D) gene expression is reduced after 4 h (grey bar) and remains at low levels at 24 h (black bar), whereas SOX10 (B) gene expression is only slightly reduced after 4 h (grey bar) but further decreases at 24 h (black bar). Experiments were performed three times in duplicates. * p<0.05, ** p<0.01, *** p<0.001 and ns for p>0.05; VN: vehicle+normoxia, LN: LPS+normoxia, VH: vehicle+hyperoxia, LH: LPS+hyperoxia.

Gene expression analysis of co-cultures after 4 and 24 h of LPS treatment indicate a loss of *Sox9* and *Sox10* which are transcriptional factors promoting oligodendrocyte formation and development (Fig. 7A: *Sox9* (4 h) 72%, p<0.05, grey bar; (24 h) 69%, p<0.01, black bar; Fig. 7B: *Sox10* (4 h) 87%, p = 0.30, grey bar; (24 h) 54%, p<0.01, black bar). Furthermore, the evaluation of markers for late immature oligodendrocytes, *CNP* and *MBP*, showed reduced gene expression after LPS treatment (Fig. 7C: *CNP* (4 h) 57%, p<0.001, grey bar; (24 h) 54%, p<0.001, black bar; Fig. 7D: *MBP* (4 h) 29%, p<0.001, grey bar; (24 h) 35%, p<0.001, black bar).

## Discussion

Inflammatory conditions are known to be relevant in inducing premature birth and are involved in causing brain damage of these infants. Since we recently confirmed that high oxygen levels contribute to neonatal brain injury, we were interested in the interaction of inflammation and hyperoxia. In a rat model, we demonstrate that systemic inflammation as well as exposure to high oxygen levels induces caspase-3 activation in whole brain homogenates. However, a more detailed analysis focusing on oligodendrocytes and distinct brain regions of white matter damage revealed a specific oligodendrocyte apoptotic cell death by hyperoxia but not by LPS. Interestingly, the number of immature oligodendrocytes (O4-positive cells) stays constant, while much fewer mature oligodendrocytes (APC-CC1-positive cells) were detected, suggesting rather an impaired maturation by LPS instead of apoptosis induction. In accordance with our results, it was demonstrated that caspase-3 activation can be related to non-apoptotic functions, i.g. reactive astrogliosis, cytoskeleton remodeling and infiltration of macrophages [51], [52]. Moreover, in another model of neonatal systemic inflammation using low dose IL-1β over a five day period, cell death was not detected. In line with our observations an imbalance between transcription factors controlling oligodendrocyte maturation resulting in a loss of mature oligodendrocytes was found [22]. After hyperoxia exposure cell death is the predominant form of injury. The number of TUNEL-positive oligodendrocytes is strongly increased resulting in a reduced amount of immature and mature oligodendrocytes. Cell death of oligodendrocyte progenitors has been reported before as a main consequence of hyperoxia induced brain injury in mice exposed to 80% oxygen for 48 h [11]. A loss of immature oligodendrocytes in rats caused by apoptotic cell death resulting in a decreased number of mature oligodendrocytes and hypomyelination was also reported after exposure to 60% oxygen from E21 to P7 [53]. These cellular damages correlate well with a marked hypomyelination induced by both noxious stimuli, indicating that oligodendrocytes, the solely myelinating cells of the CNS, are a major target in neonatal brain injury [54]. This hypomyelination occurs in comparable intensities in the single and two-hit scenarios. A time matched application of LPS and hyperoxia reveals a similar degree of hypomyelination.

In the two-hit scenario, where rat pups were treated with LPS three days prior to hyperoxia exposure at P6, myelination was reduced compared to control animals. However, no significant differences in the protein content at P11 and microstructure at P21 was detected between all treatment groups. Diffusion tensor imaging provides strong evidence that white matter injury is likely to be related to sustained white matter alteration. Indeed, according to fractional anisotropy, white matter damage is more defined at P21 than at P11, whereas MBP expression is compensated at P21 in all groups. These abnormities in white matter organization, detected by reduced fractional anisotropy, have been shown to persist into adulthood (P60) in mice, which were exposed for 48 h to 80% oxygen [11]. Similarly to our results, the authors also report an early compensation of MBP expression at P15 despite persisting structural white matter disturbances in later life. Although we detected lower cell death rates in the two-hit scenario than in hyperoxia exposed pups, neither sensitizing or protective effects of LPS pre-treatment were present in hyperoxia-induced brain injury with respect to myelination and white matter microstructure.

A retrospective study of cases with diffuse WMD has shown that the mechanism of myelination failure involves a disrupted cellular response. Oligodendrocyte progenitor cells failed to differentiate in diffuse astrogliotic lesions without decimating the total oligodendrocyte population [55]. Consequently, apoptosis is presumably not the key pathway involved in LPS-induced hypomyelination. More than 1500 genes were found to be regulated after a systemic LPS application in the developing rat brain using microarray technique [56]. Genes responsible for inflammatory response and cell death related genes were affected as well as genes regulating cell proliferation and differentiation. Pang and coworkers found an impaired development of immature oligodendrocytes by reduced number of O4 and O1 stained cells after intracerebral LPS injection [24]. Beside oligodendrocyte death through pro-inflammatory cytokines and ROS/RNS, an apoptosis-independent pathway, namely oligodendrocyte maturity arrest, is suggested to be part of LPS-induced hypomyelination [21], [57]. In addition, myelination requires electrical and molecular interactions between healthy axons and oligodendrocytes [58]. Altered WM microstructure defined by reduced fractional anisotropy at the early stage is likely to participate by impairment of the oligodendrocyte-axon interactions needed for adequate myelination process [59].

The susceptibility of the neonatal rodent brain to noxious stimuli is maturation dependent and most sensitive in the first week of life [10], [33]. This period is characterized as the phase of brain growth spurt and is comparable to an infant in the last trimester of gestation [60]. This window of vulnerability coincides with the presence of late oligodendrocyte progenitor cells [61], and infants born in this period are at high risk to develop adverse neurological outcomes [54], [62]–[64]. Additionally, a crucial role for microglia-involvement in adult and neonatal neuroinflammation with altered myelination-processes has been known for several years [65], [66]. Microglia cells but not oligodendrocytes do express toll-like receptor 4, which is triggering LPS induced-brain injury [67]. In order to investigate the effect of LPS pre-treatment on hyperoxia induced pre-oligodendrocyte cell death, we made use of an oligodendrocyte-microglia-co-culture-model. When pre-oligodendrocytes were co-cultured with microglia cells and incubated with 1 µg/ml LPS 24 h before hyperoxia exposure, we found a distinct tolerance effect *in vitro* assessed by LDH release. LPS stimulated co-cultures with or without hyperoxia treatment showed changes in cell death and significant morphology as signs for a disturbed maturation. In contrast to LPS hyperoxia exposed co-cultures show high cell death rates as well as morphological changes. However, dying cells lose their processes suggesting that the observed morphological changes in hyperoxia exposed co-cultures might be rather cell death related and less likely a sign of maturity arrest. As already shown *in vivo* we observed the same tolerance effect in co-cultures, which were stimulated with LPS and exposed to hyperoxia at the same point in time. It was described that a LPS concentration of 1 ng/ml is sufficient to induce oligodendrocyte death if microglia are present, but in this study the LPS incubation time was 120 h [67] instead of 32 h as in our study. In agreement with our findings, a slightly reduced oligodendrocyte survival was reported after incubation with LPS-activated-microglia-conditioned media (1 µg/ml) after 48 h incubation, which was more prominent after 72 h, also a high reduction in the number and length of cell processes was observed in this setting [21].

Interested in the mechanism probably responsible for the reduced oligodendrocyte susceptibility against hyperoxia, gene expression analysis of LPS stimulated co-cultures was performed. At the beginning of inflammation high amounts of *TNFα* and *IL-1β* are expressed. Both cytokines are known to influence oligodendrocyte maturation and death [16], [21]. During LPS pre-incubation transcription factors regulating oligodendrocyte development (*Sox9* and *Sox10*) and oligodendrocyte maturation markers (*CNP* and *MBP*) are decreased. However, pro-inflammatory gene expression declined at 24 h, whereas gene expression of the anti-inflammatory cytokine *IL-10* and the reactive oxygen species scavenger *SOD2* increased over time. This temporal gene expression profile suggests a compensation of pro-inflammatory mechanisms at acute points in time while anti-inflammatory, i.e. protective processes are dominating in the later time course. The anti-inflammatory properties of IL-10 provide protection of the developing white matter against inflammatory injury [68], [69]. Protective effects of exogenous SOD against hyperoxia were demonstrated *in vitro* [33]. Overexpression of SOD2 in developing olidogendrocytes was associated with decreased cell death induced by mild cystine deprivation [70]. The increased expression of these beneficial molecules may be responsible for the tolerance-effect detected in vivo and *in vitro*.

The effect of LPS pre-treatment before a second insult is highly discussed in other injury models of the developing brain. It has been shown that the application of low doses LPS can result in tolerance when given 4 to 72 h before hypoxic-ischemic insult [34], [71], [72]. This preconditioning effect is also present in adult rodents in models of stroke [73], [74] and trauma [75]. I*n vivo* as well as *in vitro*, a LPS-induced tolerance effect with regard to cell death was detected. However, this protection goes along with maturity arrest of oligodendrocytes and revealed no benefit on the phenotype, as depicted by a disturbed white matter development *in vivo*. Nevertheless, neonatal LPS pre-treatment can also mediate sensitizing effects as previously demonstrated in hypoxic-ischemia [35], [76], [77] and excitotoxicity [23] brain injury models. These studies indicate that the effect of LPS pre-treatment is highly dependent on LPS dosage and the time interval between LPS application and a second hit [38], [78]. Comparing the results derived from the sequential treatment with the simultaneous exposure to both noxious stimuli, i.e. LPS and hyperoxia, we did not detect substantial differences between both treatment strategies within our model of hyperoxia induced brain injury indicated by MBP expression and white matter microstructure.

In summary, our data suggest that inflammation as well as hyperoxia strongly attenuate oligodendrocyte cellular dynamics involving apoptosis and developmental arrest. Both, oligodendrocyte death and developmental arrest result in hypomyelination and a disturbed white matter microstructure. From a pharmacological point of view, strategies for improvement of oligodendrocyte maturation in addition to anti-apoptotic drugs might be a beneficial tool avoiding extensive WMD. The role of other presumably involved cells, like astrocytes and infiltrating immune cells, in terms of damage or repair has to be determined in further studies. The knowledge about interactions among inflammation and hyperoxia contributes to a better understanding of WMD in premature born infants and might open new access to prevention strategies.

## Supporting Information

Figure S1 **MBP expression is compensated at P21.** The reduced MBP expression found at P11 is restored 10 days later in all treated groups. (A) Results of densitometric western blot quantification of P3 LPS P6 hyperoxia experiment (A) and a representative western blot series (B) are shown.(n = 4). Ns: p>0.05; VN: vehicle+normoxia, LN: LPS+normoxia, VH: vehicle+hyperoxia, LH: LPS+hyperoxia.
(TIF)

Figure S2 **Simultanous exposure to LPS and hyperoxia reduces pre-OL susceptibility towards hyperoxia induced cell death.** Oligodendrocyte-microglia-co-cultures were exposed to hyperoxia and stimulated with LPS at the same time for 8 h. During this short period LPS did not induce any LDH release (LN, grey bar), whereas hyperoxia exposure results in an intense increase in LDH release (VH, black bar). The reduced susceptibility of oligodendrocytes after LPS pre-treatment exists during time matched exposure (LH, dashed bar). Five independent experiments were used for data collection and means+SEM were shown. *** p<0.001 and ns for p>0.5; VN: vehicle+normoxia, LN: LPS+normoxia, VH: vehicle+hyperoxia, LH: LPS+hyperoxia.
(TIF)
