# Supplementary figures and images for: Interaction of Inflammation and Hyperoxia in a Rat Model of Neonatal White Matter Damage
Source: PLoS One. 2012 Nov 14;7(11):e49023. doi: 10.1371/journal.pone.0049023 (PMC3498343; doi:10.1371/journal.pone.0049023)

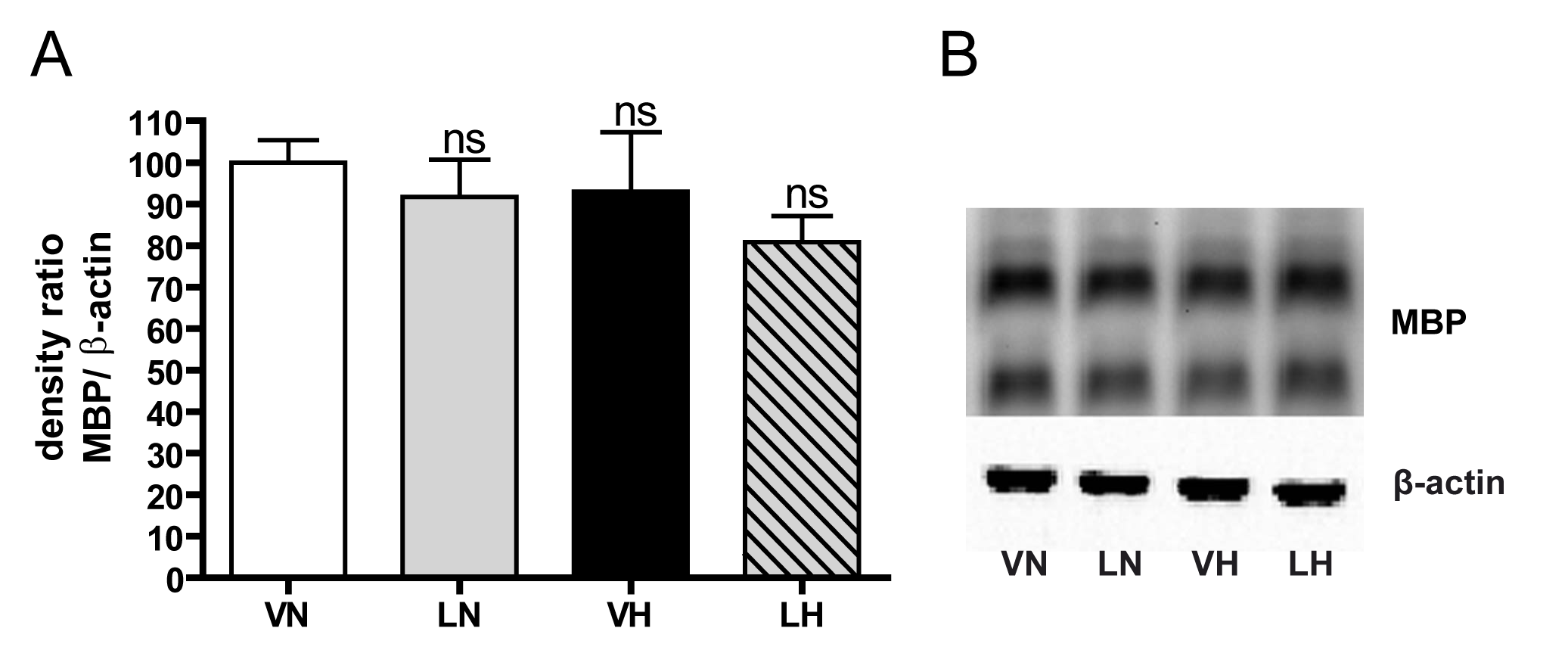

Supplement: Figure S1 — MBP expression is compensated at P21. The reduced MBP expression found at P11 is restored 10 days later in all treated groups. (A) Results of densitometric western blot quantification of P3 LPS P6 hyperoxia experiment (A) and a representative western blot series (B) are shown.(n = 4). Ns: p>0.05; VN: vehicle+normoxia, LN: LPS+normoxia, VH: vehicle+hyperoxia, LH: LPS+hyperoxia. (TIF) [file pone.0049023.s001.tif]

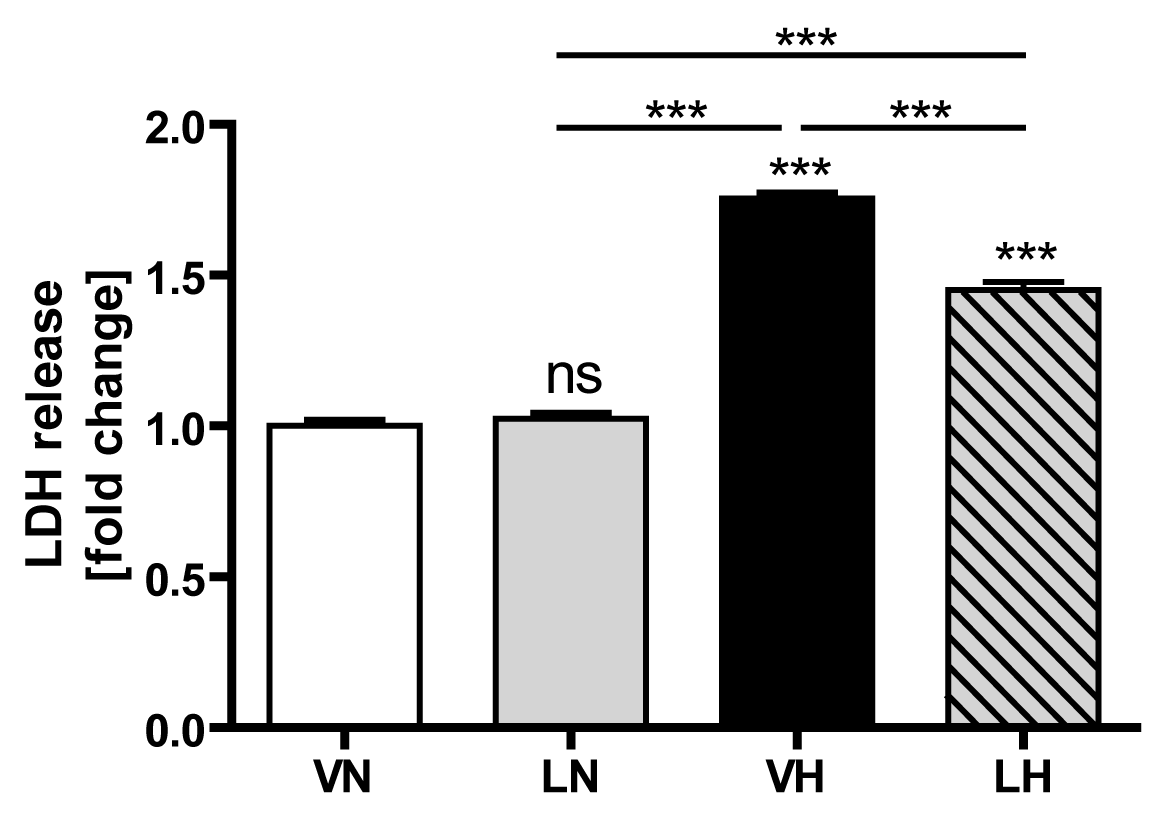

Supplement: Figure S2 — Simultanous exposure to LPS and hyperoxia reduces pre-OL susceptibility towards hyperoxia induced cell death. Oligodendrocyte-microglia-co-cultures were exposed to hyperoxia and stimulated with LPS at the same time for 8 h. During this short period LPS did not induce any LDH release (LN, grey bar), whereas hyperoxia exposure results in an intense increase in LDH release (VH, black bar). The reduced susceptibility of oligodendrocytes after LPS pre-treatment exists during time matched exposure (LH, dashed bar). Five independent experiments were used for data collection and means+SEM were shown. *** p<0.001 and ns for p>0.5; VN: vehicle+normoxia, LN: LPS+normoxia, VH: vehicle+hyperoxia, LH: LPS+hyperoxia. (TIF) [file pone.0049023.s002.tif]
